# Bis(ferrocenecarbaldehyde thio­semi­carbazonato-κ^2^ *N* ^1^,*S*)zinc

**DOI:** 10.1107/S1600536809007363

**Published:** 2009-03-06

**Authors:** M. R. Vikneswaran, Siang Guan Teoh, Ibrahim Abdul Razak, Hoong-Kun Fun

**Affiliations:** a School of Chemical Sciences, Universiti Sains Malaysia, 11800 USM, Penang, Malaysia; b X-ray Crystallography Unit, School of Physics, Universiti Sains Malaysia, 11800 USM, Penang, Malaysia

## Abstract

In the title compound, [Fe_2_Zn(C_5_H_5_)_2_(C_7_H_7_N_3_S)_2_], the Cp rings of each ferrocene residue have a nearly eclipsed conformation. The two thio­semicarbazone ligands each coordinate the Zn atom in a bidentate mode *via* the N and S atoms, thereby defining a distorted tetra­hedral environment. N—H⋯S, N—H⋯N, C—H⋯S and C—H⋯N intra- and intermol­ecular inter­actions connect the mol­ecules into a two-dimensional array parallel to (010).

## Related literature

For general background, see: Quiroga *et al.* (1998 ▶); Genova *et al.* (2004 ▶); Melha (2008 ▶). For related structures, see: Palenik (1970 ▶); Haaland & Sikson (1968 ▶); Li *et al.* (2004 ▶); Latheef *et al.* (2007 ▶). For the synthesis, see: Mariño *et al.* (2006 ▶). For the stability of the temperature controller, see: Cosier & Glazer (1986 ▶).
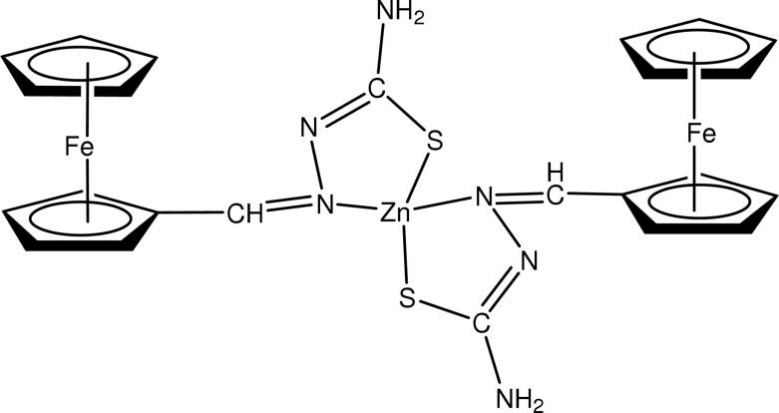


## Experimental

### 

#### Crystal data

[Fe_2_Zn(C_5_H_5_)_2_(C_7_H_7_N_3_S)_2_]
*M* *_r_* = 637.68
Monoclinic, 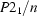

*a* = 10.8483 (2) Å
*b* = 14.7547 (2) Å
*c* = 16.1686 (2) Å
β = 105.252 (1)°
*V* = 2496.85 (6) Å^3^
*Z* = 4
Mo *K*α radiation
μ = 2.29 mm^−1^
*T* = 100 K
0.58 × 0.19 × 0.08 mm

#### Data collection

Bruker SMART CCD area-detector diffractometer
Absorption correction: multi-scan (*SADABS*; Bruker, 2005 ▶) *T*_min_ = 0.348, *T*_max_ = 0.840
61395 measured reflections
11266 independent reflections
7721 reflections with *I* > 2σ(*I*)
*R*_int_ = 0.052

#### Refinement

*R*[*F* ^2^ > 2σ(*F* ^2^)] = 0.042
*wR*(*F* ^2^) = 0.010
*S* = 1.06
11266 reflections
332 parameters
1 restraint
H atoms treated by a mixture of independent and constrained refinement
Δρ_max_ = 0.99 e Å^−3^
Δρ_min_ = −0.64 e Å^−3^

### 

Data collection: *SMART* (Bruker, 2005 ▶); cell refinement: *SAINT* (Bruker, 2005 ▶); data reduction: *SAINT*; program(s) used to solve structure: *SHELXTL* (Sheldrick, 2008 ▶); program(s) used to refine structure: *SHELXTL*; molecular graphics: *SHELXTL*; software used to prepare material for publication: *SHELXTL* and *PLATON* (Spek, 2009 ▶).

## Supplementary Material

Crystal structure: contains datablocks global, I. DOI: 10.1107/S1600536809007363/tk2381sup1.cif

Structure factors: contains datablocks I. DOI: 10.1107/S1600536809007363/tk2381Isup2.hkl

Additional supplementary materials:  crystallographic information; 3D view; checkCIF report

## Figures and Tables

**Table 1:** Hydrogen-bond geometry (Å, °)

*D*—H⋯*A*	*D*—H	H⋯*A*	*D*⋯*A*	*D*—H⋯*A*
N5—H2*N*5⋯S2^i^	0.80 (3)	2.80 (2)	3.4880 (19)	146 (2)
N6—H2*N*6⋯N2^ii^	0.81 (3)	2.21 (3)	2.983 (2)	161 (3)
C7—H7*A*⋯N2^iii^	0.98	2.56	3.465 (3)	153
C9—H9*A*⋯S2	0.98	2.73	3.697 (2)	168
C17—H17*A*⋯S2^iv^	0.98	2.70	3.479 (2)	137
C19—H19*A*⋯N4	0.98	2.51	2.936 (2)	106
N5—H1*N*5⋯*Cg*1^v^	0.90 (3)	2.85 (3)	3.515 (2)	132 (2)
C6—H6*A*⋯*Cg*2^vi^	0.98	2.80	3.755 (2)	164
C10—H10*A*⋯*Cg*3^vi^	0.98	2.93	3.674 (3)	134
C24—H24*A*⋯*Cg*4^iv^	0.98	2.83	3.629 (2)	139

Symmetry codes: (i) 
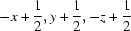
; (ii) 
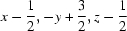
; (iii) 
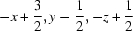
; (iv) 
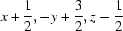
; (v) 
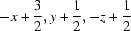
; (vi) 
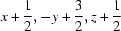
. *Cg*1, *Cg*2, *Cg*3, and *Cg*4 are the centroids of C20–C24, C15–C19, Zn1,S2,C2,N4,N3 and Zn1,S1,C1,N2,N1 rings, respectively.

**Fig. 1.**
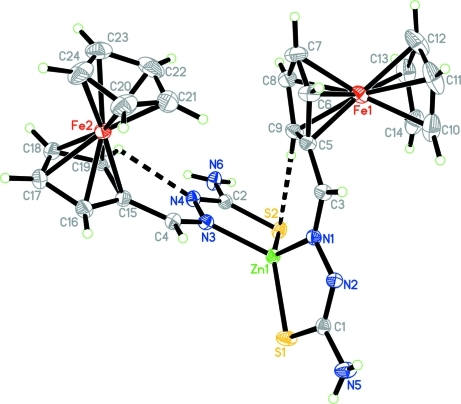
The molecular structure of (I), showing 50% probability displacement ellipsoids and the atom numbering scheme. Intramolecular contacts are drawn as dashed lines.

**Fig. 2.**
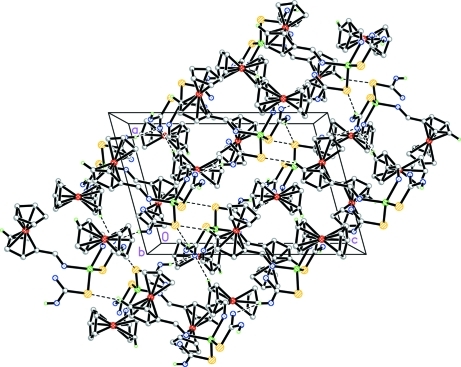
The crystal packing for (I), viewed down the b axis. H atoms excepting for those involved in intermolecular interactions (shown as dashed lines) are omitted for clarity.

## supplementary crystallographic information

### Comment

Thiosemicarbazones and their metal complexes have been attracting considerable interest due to their biological activities, such as anti-tumor (Quiroga *et al.*, 1998), anti-viral (Genova *et al.*, 2004), anti-fungal and anti-bacterial properties (Melha, 2008). Herein, we report the crystal structure of the zinc(II) complex formed with formylferrocene thiosemicarbazone, (I).

The average Fe—C_ring_ distances in (I) (Fig. 1) are as expected for a ferrocene derivative [Fe—Cp1(C5—C9) = 2.043 (2) Å, Fe—Cp2(C10—C14) = 2.050 (2) Å, Fe—Cp3(C15—C19) = 2.046 (2) Å and Fe—Cp4(C20—C24) = 2.044 (2) Å] and are within the range of the values in the structures reported by Palenik (1970) and Haaland & Sikson (1968). The torsion angle for a ring-center, a ring-C atom, the corresponding C atom on the opposite ring and its ring center defines the angle of twist of the Cp rings (Palenik, 1970). The average twist angles are 5.4 and 7.7° for Cp1/Cp2 and Cp3/Cp4, respectively, indicating both Cp rings adopt nearly eclipsed conformations. Each of the Cp1/Cp2 and Cp3/Cp4 pairs are nearly parallel [dihedral angle for Cp1/Cp2 = 0.68 (13)° and 1.02 (13)° for Cp3/Cp4] and co-planar [maximum deviation for Cp1 is 0.006 (2)Å for atom C5; for Cp2 is 0.002 (2)Å for atom C13; for Cp3 is 0.005 (2)Å for both atoms C15 and C16; and for Cp4 is 0.004 (3)Å for both atoms C20 and C21].

Each of the two thiosemicarbazone ligands is bidentately coordinated to Zn(II) *via* the N and S atoms thus forming a distorted tetrahedral environment. The angles around Zn atom ranges from 86.58 (4) to 131.68 (4)°. These chelating ligands form two five membered rings, Zn1S1C1N1N2 and Zn1S2C2N3N4, which are planar with maximum deviation of 0.011 (1) and 0.014 (1) Å, respectively for the Zn atom. The two five-membered rings form a dihedral angle of 77.75 (5)°. Distorted tetrahedral coordination for Zn(II) was also reported by Li *et al.* (2004) and Latheef *et al.* (2007). Bond lengths involving the thiosemicarbazone moieties are generally comparable to the corresponding values reported by Latheef *et al.* (2007) with the exception of C1—N2 which is shorter and N1—N2, Zn1—S2 and C2—S2 which are longer. This observation is probably due to atoms N2 and S2 being involved in intermolecular interactions (Table 1).

The crystal structure is stabilized by N—H···S, N—H···N, C—H···S and C—H···N intermolecular interactions (Table 1). These interactions link the molecules into 2-D arrays parallel to the *ac* plane (Fig. 2). The crystal structure is further stabilized by the N—H···π and C—H···π interactions (Table 1).

### Experimental

Formylferrocene thiosemicarbazone was prepared as described by Mariño *et al.* (2006). Zn(CH_3_COO)_2_.2H_2_O(0.21 g,1 mmol) dissolved in methanol (60 ml) was added dropwise at room temperature to a mixture of formylferrocene thiosemicarbazone (0.28 g, 1 mmol) and KOH (0.12 g, 2 mmol) in absolute methanol (15 ml). Amorphous orange solids separated out immediately. The suspension was stirred under reflux for 4 h and filtered. After several days, brown crystals were obtained from the filtrate.

### Refinement

All the H atoms were positioned geometrically and refined using a riding model with C—H = 0.93–0.98 Å, and with U_iso_(H) = 1.2 U_eq_ (C) The N-bound H atoms were located from a difference Fourier map and refined isotropically with N-H = 0.779 (15) - 0.90 (3) Å.

### Crystal data

**Table:**

[Fe_2_Zn(C_5_H_5_)_2_(C_7_H_7_N_3_S)_2_]	*F*(000) = 1296
*M**_r_* = 637.68	*D*_x_ = 1.696 Mg m^−^^3^
Monoclinic, *P*2_1_/*n*	Mo *K*α radiation, λ = 0.71073 Å
Hall symbol: -P 2yn	Cell parameters from 9980 reflections
*a* = 10.8483 (2) Å	θ = 2.4–34.0°
*b* = 14.7547 (2) Å	µ = 2.29 mm^−^^1^
*c* = 16.1686 (2) Å	*T* = 100 K
β = 105.252 (1)°	Plate, brown
*V* = 2496.85 (6) Å^3^	0.58 × 0.19 × 0.08 mm
*Z* = 4	

### Data collection

**Table:**

Bruker SMART CCD area-detector diffractometer	11266 independent reflections
Radiation source: sealed tube	7721 reflections with *I* > 2σ(*I*)
graphite	*R*_int_ = 0.052
π and ω scans	θ_max_ = 35.4°, θ_min_ = 1.9°
Absorption correction: multi-scan (*SADABS*; Bruker, 2005)	*h* = −17→16
*T*_min_ = 0.348, *T*_max_ = 0.840	*k* = −21→23
61395 measured reflections	*l* = −26→26

### Refinement

**Table:**

Refinement on *F*^2^	Primary atom site location: structure-invariant direct methods
Least-squares matrix: full	Secondary atom site location: difference Fourier map
*R*[*F*^2^ > 2σ(*F*^2^)] = 0.042	Hydrogen site location: inferred from neighbouring sites
*wR*(*F*^2^) = 0.010	H atoms treated by a mixture of independent and constrained refinement
*S* = 1.06	*w* = 1/[σ^2^(*F*_o_^2^) + (0.0428*P*)^2^ + 0.3689*P*] where *P* = (*F*_o_^2^ + 2*F*_c_^2^)/3
11266 reflections	(Δ/σ)_max_ = 0.002
332 parameters	Δρ_max_ = 0.99 e Å^−^^3^
1 restraint	Δρ_min_ = −0.64 e Å^−^^3^

### Special details

**Table:**

Experimental. The crystal was placed in the cold stream of an Oxford Cryosystems Cobra open-flow nitrogen cryostat (Cosier & Glazer, 1986) operating at 100.0 (1) K.
Geometry. All e.s.d.'s (except the e.s.d. in the dihedral angle between two l.s. planes) are estimated using the full covariance matrix. The cell e.s.d.'s are taken into account individually in the estimation of e.s.d.'s in distances, angles and torsion angles; correlations between e.s.d.'s in cell parameters are only used when they are defined by crystal symmetry. An approximate (isotropic) treatment of cell e.s.d.'s is used for estimating e.s.d.'s involving l.s. planes.
Refinement. Refinement of *F*^2^ against ALL reflections. The weighted *R*-factor *wR* and goodness of fit *S* are based on *F*^2^, conventional *R*-factors *R* are based on *F*, with *F* set to zero for negative *F*^2^. The threshold expression of *F*^2^ > σ(*F*^2^) is used only for calculating *R*-factors(gt) *etc*. and is not relevant to the choice of reflections for refinement. *R*-factors based on *F*^2^ are statistically about twice as large as those based on *F*, and *R*- factors based on ALL data will be even larger.

### Fractional atomic coordinates and isotropic or equivalent isotropic displacement parameters (Å^2^)

**Table:**

	*x*	*y*	*z*	*U*_iso_*/*U*_eq_	
Zn1	0.360473 (19)	0.818097 (14)	0.175658 (12)	0.01591 (5)	
Fe1	0.60392 (3)	0.553115 (18)	0.319621 (17)	0.02062 (7)	
Fe2	0.65545 (3)	0.713991 (19)	−0.064830 (17)	0.02060 (7)	
S1	0.33717 (5)	0.96352 (3)	0.21462 (3)	0.02417 (11)	
S2	0.19130 (4)	0.72073 (3)	0.14688 (3)	0.02045 (10)	
N6	0.13760 (17)	0.62297 (12)	0.00529 (11)	0.0229 (3)	
C1	0.48096 (17)	0.96209 (12)	0.29426 (11)	0.0173 (3)	
C2	0.21813 (17)	0.68618 (12)	0.04915 (11)	0.0179 (3)	
C3	0.62251 (17)	0.75580 (12)	0.28741 (11)	0.0190 (3)	
H3A	0.6967	0.7718	0.3286	0.023*	
C4	0.47629 (17)	0.81122 (12)	0.02822 (12)	0.0188 (3)	
H4A	0.5304	0.8539	0.0616	0.023*	
C5	0.61764 (18)	0.66613 (12)	0.25081 (12)	0.0198 (4)	
C6	0.72976 (19)	0.61046 (13)	0.26228 (13)	0.0252 (4)	
H6A	0.8176	0.6286	0.2907	0.030*	
C7	0.6908 (2)	0.52516 (14)	0.22429 (15)	0.0304 (5)	
H7A	0.7471	0.4735	0.2231	0.037*	
C8	0.5564 (2)	0.52614 (14)	0.19008 (13)	0.0263 (4)	
H8A	0.5041	0.4752	0.1616	0.032*	
C9	0.50977 (19)	0.61241 (13)	0.20639 (12)	0.0232 (4)	
H9A	0.4203	0.6319	0.1903	0.028*	
C10	0.5945 (3)	0.59366 (17)	0.43946 (14)	0.0393 (6)	
H10A	0.6008	0.6563	0.4604	0.047*	
C11	0.6971 (3)	0.53416 (18)	0.44599 (15)	0.0451 (7)	
H11A	0.7872	0.5478	0.4727	0.054*	
C12	0.6479 (2)	0.44998 (17)	0.40671 (16)	0.0417 (6)	
H12A	0.6977	0.3956	0.4023	0.050*	
C13	0.5132 (2)	0.45957 (15)	0.37643 (14)	0.0343 (5)	
H13A	0.4533	0.4130	0.3468	0.041*	
C14	0.4806 (3)	0.54866 (16)	0.39660 (15)	0.0358 (5)	
H14A	0.3943	0.5744	0.3830	0.043*	
C15	0.50287 (17)	0.78963 (13)	−0.05250 (11)	0.0187 (3)	
C16	0.58907 (19)	0.84334 (14)	−0.08659 (13)	0.0236 (4)	
H16A	0.6338	0.8981	−0.0604	0.028*	
C17	0.5977 (2)	0.80355 (14)	−0.16440 (13)	0.0266 (4)	
H17A	0.6500	0.8257	−0.2012	0.032*	
C18	0.5194 (2)	0.72454 (14)	−0.17923 (12)	0.0251 (4)	
H18A	0.5089	0.6831	−0.2280	0.030*	
C19	0.46084 (18)	0.71495 (13)	−0.11123 (12)	0.0211 (4)	
H19A	0.4025	0.6663	−0.1049	0.025*	
C20	0.8388 (2)	0.71997 (17)	0.01123 (15)	0.0353 (5)	
H20A	0.8880	0.7752	0.0308	0.042*	
C21	0.7588 (2)	0.67432 (18)	0.05388 (15)	0.0408 (6)	
H21A	0.7429	0.6921	0.1086	0.049*	
C22	0.7063 (2)	0.59671 (17)	0.00334 (17)	0.0416 (6)	
H22A	0.6478	0.5521	0.0171	0.050*	
C23	0.7539 (2)	0.59651 (16)	−0.06984 (16)	0.0370 (5)	
H23A	0.7335	0.5518	−0.1162	0.044*	
C24	0.8343 (2)	0.67154 (17)	−0.06532 (16)	0.0362 (5)	
H24A	0.8798	0.6880	−0.1081	0.043*	
N5	0.50939 (17)	1.03882 (12)	0.34266 (11)	0.0241 (4)	
N1	0.53245 (14)	0.81584 (10)	0.26777 (9)	0.0164 (3)	
N2	0.56360 (14)	0.89628 (10)	0.31495 (9)	0.0169 (3)	
N3	0.38768 (14)	0.77964 (10)	0.06093 (9)	0.0176 (3)	
N4	0.30745 (14)	0.71515 (10)	0.01374 (9)	0.0179 (3)	
H1N6	0.0846 (18)	0.6050 (15)	0.0255 (13)	0.020 (6)*	
H2N5	0.471 (2)	1.0838 (18)	0.3241 (16)	0.032 (7)*	
H1N5	0.592 (3)	1.0432 (18)	0.3714 (17)	0.046 (8)*	
H2N6	0.136 (3)	0.6177 (19)	−0.0447 (18)	0.045 (8)*	

### Atomic displacement parameters (Å^2^)

**Table:**

	*U*^11^	*U*^22^	*U*^33^	*U*^12^	*U*^13^	*U*^23^
Zn1	0.01686 (10)	0.01601 (10)	0.01412 (9)	−0.00136 (7)	0.00276 (7)	−0.00117 (7)
Fe1	0.01956 (13)	0.01766 (13)	0.02229 (13)	−0.00241 (9)	0.00134 (10)	0.00225 (10)
Fe2	0.01781 (13)	0.02438 (15)	0.02068 (13)	0.00488 (10)	0.00698 (10)	0.00279 (10)
S1	0.0223 (2)	0.0179 (2)	0.0268 (2)	0.00339 (16)	−0.00326 (18)	−0.00247 (17)
S2	0.0205 (2)	0.0233 (2)	0.0188 (2)	−0.00489 (16)	0.00733 (16)	−0.00330 (16)
N6	0.0226 (8)	0.0281 (9)	0.0190 (8)	−0.0077 (6)	0.0074 (6)	−0.0042 (6)
C1	0.0178 (8)	0.0184 (8)	0.0156 (8)	−0.0025 (6)	0.0044 (6)	−0.0005 (6)
C2	0.0167 (8)	0.0192 (8)	0.0165 (8)	0.0009 (6)	0.0023 (6)	0.0002 (6)
C3	0.0178 (8)	0.0175 (8)	0.0206 (8)	−0.0004 (6)	0.0029 (6)	0.0006 (6)
C4	0.0169 (8)	0.0201 (9)	0.0193 (8)	−0.0004 (6)	0.0047 (6)	−0.0005 (6)
C5	0.0218 (9)	0.0166 (8)	0.0211 (8)	0.0008 (6)	0.0058 (7)	0.0017 (6)
C6	0.0218 (9)	0.0191 (9)	0.0357 (11)	0.0018 (7)	0.0093 (8)	0.0012 (8)
C7	0.0336 (11)	0.0206 (10)	0.0405 (12)	0.0054 (8)	0.0159 (9)	−0.0009 (9)
C8	0.0331 (11)	0.0208 (9)	0.0244 (9)	0.0007 (8)	0.0062 (8)	−0.0024 (7)
C9	0.0249 (9)	0.0193 (9)	0.0219 (9)	0.0023 (7)	−0.0003 (7)	0.0008 (7)
C10	0.0569 (16)	0.0370 (13)	0.0239 (10)	−0.0202 (11)	0.0103 (10)	−0.0027 (9)
C11	0.0459 (14)	0.0475 (15)	0.0301 (12)	−0.0216 (12)	−0.0109 (10)	0.0161 (10)
C12	0.0434 (14)	0.0312 (12)	0.0421 (13)	−0.0072 (10)	−0.0038 (11)	0.0179 (10)
C13	0.0407 (13)	0.0312 (12)	0.0301 (11)	−0.0168 (9)	0.0075 (9)	0.0017 (9)
C14	0.0429 (14)	0.0394 (13)	0.0291 (11)	−0.0129 (10)	0.0164 (10)	−0.0049 (9)
C15	0.0176 (8)	0.0209 (9)	0.0178 (8)	0.0028 (6)	0.0047 (6)	0.0010 (6)
C16	0.0253 (10)	0.0223 (9)	0.0246 (9)	0.0011 (7)	0.0092 (7)	0.0038 (7)
C17	0.0330 (11)	0.0286 (11)	0.0214 (9)	0.0044 (8)	0.0127 (8)	0.0060 (8)
C18	0.0295 (10)	0.0263 (10)	0.0192 (9)	0.0063 (8)	0.0060 (7)	0.0001 (7)
C19	0.0187 (9)	0.0226 (9)	0.0211 (9)	0.0027 (7)	0.0034 (7)	−0.0005 (7)
C20	0.0236 (11)	0.0426 (14)	0.0359 (12)	0.0096 (9)	0.0011 (9)	−0.0089 (10)
C21	0.0372 (13)	0.0574 (16)	0.0257 (11)	0.0294 (11)	0.0044 (9)	0.0097 (10)
C22	0.0325 (12)	0.0380 (14)	0.0522 (15)	0.0138 (10)	0.0074 (11)	0.0195 (11)
C23	0.0306 (12)	0.0331 (12)	0.0440 (13)	0.0138 (9)	0.0044 (10)	−0.0020 (10)
C24	0.0204 (10)	0.0505 (15)	0.0383 (12)	0.0101 (9)	0.0089 (9)	−0.0074 (10)
N5	0.0223 (8)	0.0179 (8)	0.0286 (9)	0.0011 (6)	0.0006 (7)	−0.0042 (6)
N1	0.0187 (7)	0.0146 (7)	0.0154 (6)	−0.0017 (5)	0.0039 (5)	0.0001 (5)
N2	0.0187 (7)	0.0145 (7)	0.0176 (7)	−0.0002 (5)	0.0050 (5)	−0.0001 (5)
N3	0.0179 (7)	0.0167 (7)	0.0174 (7)	−0.0010 (5)	0.0033 (5)	−0.0011 (5)
N4	0.0176 (7)	0.0193 (7)	0.0159 (7)	−0.0021 (5)	0.0029 (5)	−0.0008 (5)

### Geometric parameters (Å, °)

**Table:**

Zn1—N3	2.0342 (16)	C6—C7	1.415 (3)
Zn1—N1	2.0586 (14)	C6—H6A	0.9800
Zn1—S1	2.2690 (5)	C7—C8	1.417 (3)
Zn1—S2	2.2802 (5)	C7—H7A	0.9800
Fe1—C6	2.026 (2)	C8—C9	1.420 (3)
Fe1—C5	2.0317 (18)	C8—H8A	0.9800
Fe1—C12	2.043 (2)	C9—H9A	0.9800
Fe1—C9	2.0436 (18)	C10—C11	1.399 (4)
Fe1—C11	2.046 (2)	C10—C14	1.414 (3)
Fe1—C13	2.048 (2)	C10—H10A	0.9800
Fe1—C7	2.049 (2)	C11—C12	1.433 (3)
Fe1—C14	2.054 (2)	C11—H11A	0.9800
Fe1—C10	2.056 (2)	C12—C13	1.420 (3)
Fe1—C8	2.060 (2)	C12—H12A	0.9800
Fe2—C21	2.037 (2)	C13—C14	1.421 (3)
Fe2—C16	2.038 (2)	C13—H13A	0.9800
Fe2—C24	2.040 (2)	C14—H14A	0.9800
Fe2—C19	2.0461 (18)	C15—C16	1.441 (3)
Fe2—C20	2.046 (2)	C15—C19	1.448 (3)
Fe2—C18	2.0465 (19)	C16—C17	1.414 (3)
Fe2—C22	2.048 (2)	C16—H16A	0.9800
Fe2—C23	2.049 (2)	C17—C18	1.425 (3)
Fe2—C15	2.0491 (18)	C17—H17A	0.9800
Fe2—C17	2.049 (2)	C18—C19	1.414 (3)
S1—C1	1.7421 (17)	C18—H18A	0.9800
S2—C2	1.7560 (19)	C19—H19A	0.9800
N6—C2	1.345 (2)	C20—C21	1.413 (4)
N6—H1N6	0.779 (15)	C20—C24	1.419 (3)
N6—H2N6	0.81 (3)	C20—H20A	0.9800
C1—N2	1.304 (2)	C21—C22	1.435 (4)
C1—N5	1.365 (2)	C21—H21A	0.9800
C2—N4	1.320 (2)	C22—C23	1.410 (4)
C3—N1	1.295 (2)	C22—H22A	0.9800
C3—C5	1.445 (3)	C23—C24	1.399 (4)
C3—H3A	0.9300	C23—H23A	0.9800
C4—N3	1.299 (2)	C24—H24A	0.9800
C4—C15	1.444 (3)	N5—H2N5	0.80 (3)
C4—H4A	0.9300	N5—H1N5	0.90 (3)
C5—C6	1.439 (3)	N1—N2	1.403 (2)
C5—C9	1.439 (3)	N3—N4	1.377 (2)
			
N3—Zn1—N1	109.51 (6)	C7—C6—H6A	126.1
N3—Zn1—S1	124.78 (5)	C5—C6—H6A	126.1
N1—Zn1—S1	87.73 (4)	Fe1—C6—H6A	126.1
N3—Zn1—S2	86.58 (4)	C6—C7—C8	108.63 (18)
N1—Zn1—S2	131.68 (4)	C6—C7—Fe1	68.81 (12)
S1—Zn1—S2	120.66 (2)	C8—C7—Fe1	70.23 (12)
C6—Fe1—C5	41.53 (8)	C6—C7—H7A	125.7
C6—Fe1—C12	124.29 (10)	C8—C7—H7A	125.7
C5—Fe1—C12	162.08 (9)	Fe1—C7—H7A	125.7
C6—Fe1—C9	69.48 (8)	C7—C8—C9	108.46 (18)
C5—Fe1—C9	41.36 (7)	C7—C8—Fe1	69.41 (12)
C12—Fe1—C9	155.16 (9)	C9—C8—Fe1	69.14 (11)
C6—Fe1—C11	107.38 (9)	C7—C8—H8A	125.8
C5—Fe1—C11	124.81 (9)	C9—C8—H8A	125.8
C12—Fe1—C11	41.02 (10)	Fe1—C8—H8A	125.8
C9—Fe1—C11	162.19 (10)	C8—C9—C5	107.70 (17)
C6—Fe1—C13	161.43 (9)	C8—C9—Fe1	70.38 (11)
C5—Fe1—C13	155.78 (9)	C5—C9—Fe1	68.88 (10)
C12—Fe1—C13	40.64 (10)	C8—C9—H9A	126.1
C9—Fe1—C13	120.30 (9)	C5—C9—H9A	126.1
C11—Fe1—C13	68.25 (9)	Fe1—C9—H9A	126.1
C6—Fe1—C7	40.64 (8)	C11—C10—C14	108.5 (2)
C5—Fe1—C7	68.82 (8)	C11—C10—Fe1	69.70 (14)
C12—Fe1—C7	107.36 (11)	C14—C10—Fe1	69.82 (13)
C9—Fe1—C7	68.45 (8)	C11—C10—H10A	125.7
C11—Fe1—C7	121.29 (11)	C14—C10—H10A	125.7
C13—Fe1—C7	124.91 (9)	Fe1—C10—H10A	125.7
C6—Fe1—C14	156.25 (9)	C10—C11—C12	108.3 (2)
C5—Fe1—C14	120.75 (9)	C10—C11—Fe1	70.41 (13)
C12—Fe1—C14	68.26 (11)	C12—C11—Fe1	69.35 (13)
C9—Fe1—C14	107.75 (10)	C10—C11—H11A	125.8
C11—Fe1—C14	67.67 (11)	C12—C11—H11A	125.8
C13—Fe1—C14	40.55 (10)	Fe1—C11—H11A	125.8
C7—Fe1—C14	162.02 (9)	C13—C12—C11	107.2 (2)
C6—Fe1—C10	121.07 (9)	C13—C12—Fe1	69.87 (13)
C5—Fe1—C10	107.83 (9)	C11—C12—Fe1	69.64 (13)
C12—Fe1—C10	68.13 (11)	C13—C12—H12A	126.4
C9—Fe1—C10	125.65 (10)	C11—C12—H12A	126.4
C11—Fe1—C10	39.88 (11)	Fe1—C12—H12A	126.4
C13—Fe1—C10	67.95 (9)	C12—C13—C14	108.0 (2)
C7—Fe1—C10	156.08 (10)	C12—C13—Fe1	69.49 (13)
C14—Fe1—C10	40.24 (9)	C14—C13—Fe1	69.97 (13)
C6—Fe1—C8	68.53 (8)	C12—C13—H13A	126.0
C5—Fe1—C8	68.69 (8)	C14—C13—H13A	126.0
C12—Fe1—C8	120.50 (10)	Fe1—C13—H13A	126.0
C9—Fe1—C8	40.48 (8)	C10—C14—C13	108.0 (2)
C11—Fe1—C8	156.15 (11)	C10—C14—Fe1	69.95 (14)
C13—Fe1—C8	107.73 (9)	C13—C14—Fe1	69.49 (14)
C7—Fe1—C8	40.36 (9)	C10—C14—H14A	126.0
C14—Fe1—C8	125.53 (9)	C13—C14—H14A	126.0
C10—Fe1—C8	162.43 (10)	Fe1—C14—H14A	126.0
C21—Fe2—C16	120.90 (9)	C16—C15—C4	121.88 (17)
C21—Fe2—C24	68.13 (10)	C16—C15—C19	106.97 (17)
C16—Fe2—C24	125.45 (10)	C4—C15—C19	131.09 (18)
C21—Fe2—C19	127.32 (10)	C16—C15—Fe2	68.92 (11)
C16—Fe2—C19	69.30 (8)	C4—C15—Fe2	124.50 (13)
C24—Fe2—C19	153.00 (9)	C19—C15—Fe2	69.19 (10)
C21—Fe2—C20	40.49 (11)	C17—C16—C15	108.26 (18)
C16—Fe2—C20	107.86 (9)	C17—C16—Fe2	70.21 (12)
C24—Fe2—C20	40.63 (9)	C15—C16—Fe2	69.78 (11)
C19—Fe2—C20	164.97 (9)	C17—C16—H16A	125.9
C21—Fe2—C18	163.86 (11)	C15—C16—H16A	125.9
C16—Fe2—C18	68.57 (8)	Fe2—C16—H16A	125.9
C24—Fe2—C18	118.71 (9)	C16—C17—C18	108.26 (19)
C19—Fe2—C18	40.42 (8)	C16—C17—Fe2	69.31 (11)
C20—Fe2—C18	153.60 (10)	C18—C17—Fe2	69.53 (11)
C21—Fe2—C22	41.12 (11)	C16—C17—H17A	125.9
C16—Fe2—C22	156.54 (10)	C18—C17—H17A	125.9
C24—Fe2—C22	67.73 (10)	Fe2—C17—H17A	125.9
C19—Fe2—C22	108.00 (9)	C19—C18—C17	108.71 (18)
C20—Fe2—C22	68.37 (10)	C19—C18—Fe2	69.77 (10)
C18—Fe2—C22	125.37 (10)	C17—C18—Fe2	69.75 (11)
C21—Fe2—C23	68.33 (10)	C19—C18—H18A	125.6
C16—Fe2—C23	161.78 (10)	C17—C18—H18A	125.6
C24—Fe2—C23	40.01 (10)	Fe2—C18—H18A	125.6
C19—Fe2—C23	119.33 (9)	C18—C19—C15	107.79 (17)
C20—Fe2—C23	68.03 (9)	C18—C19—Fe2	69.80 (11)
C18—Fe2—C23	106.76 (9)	C15—C19—Fe2	69.41 (10)
C22—Fe2—C23	40.27 (10)	C18—C19—H19A	126.1
C21—Fe2—C15	108.85 (8)	C15—C19—H19A	126.1
C16—Fe2—C15	41.30 (8)	Fe2—C19—H19A	126.1
C24—Fe2—C15	163.88 (9)	C21—C20—C24	107.5 (2)
C19—Fe2—C15	41.40 (7)	C21—C20—Fe2	69.41 (13)
C20—Fe2—C15	126.89 (8)	C24—C20—Fe2	69.46 (13)
C18—Fe2—C15	68.74 (8)	C21—C20—H20A	126.2
C22—Fe2—C15	121.20 (9)	C24—C20—H20A	126.2
C23—Fe2—C15	155.18 (9)	Fe2—C20—H20A	126.2
C21—Fe2—C17	154.70 (10)	C20—C21—C22	107.8 (2)
C16—Fe2—C17	40.47 (8)	C20—C21—Fe2	70.10 (13)
C24—Fe2—C17	106.82 (10)	C22—C21—Fe2	69.84 (13)
C19—Fe2—C17	68.58 (8)	C20—C21—H21A	126.1
C20—Fe2—C17	119.49 (10)	C22—C21—H21A	126.1
C18—Fe2—C17	40.72 (8)	Fe2—C21—H21A	126.1
C22—Fe2—C17	162.00 (10)	C23—C22—C21	107.5 (2)
C23—Fe2—C17	124.70 (9)	C23—C22—Fe2	69.90 (13)
C15—Fe2—C17	68.74 (8)	C21—C22—Fe2	69.05 (13)
C1—S1—Zn1	92.88 (6)	C23—C22—H22A	126.2
C2—S2—Zn1	92.95 (6)	C21—C22—H22A	126.2
C2—N6—H1N6	117.6 (16)	Fe2—C22—H22A	126.2
C2—N6—H2N6	116 (2)	C24—C23—C22	108.4 (2)
H1N6—N6—H2N6	124 (3)	C24—C23—Fe2	69.67 (13)
N2—C1—N5	115.74 (16)	C22—C23—Fe2	69.82 (13)
N2—C1—S1	128.32 (14)	C24—C23—H23A	125.8
N5—C1—S1	115.92 (14)	C22—C23—H23A	125.8
N4—C2—N6	116.42 (17)	Fe2—C23—H23A	125.8
N4—C2—S2	127.65 (14)	C23—C24—C20	108.8 (2)
N6—C2—S2	115.93 (15)	C23—C24—Fe2	70.32 (13)
N1—C3—C5	125.60 (16)	C20—C24—Fe2	69.91 (13)
N1—C3—H3A	117.2	C23—C24—H24A	125.6
C5—C3—H3A	117.2	C20—C24—H24A	125.6
N3—C4—C15	129.32 (17)	Fe2—C24—H24A	125.6
N3—C4—H4A	115.3	C1—N5—H2N5	117.2 (18)
C15—C4—H4A	115.3	C1—N5—H1N5	113.6 (18)
C6—C5—C9	107.38 (16)	H2N5—N5—H1N5	119 (3)
C6—C5—C3	122.10 (17)	C3—N1—N2	112.77 (14)
C9—C5—C3	130.25 (17)	C3—N1—Zn1	132.08 (12)
C6—C5—Fe1	69.03 (11)	N2—N1—Zn1	114.99 (10)
C9—C5—Fe1	69.77 (11)	C1—N2—N1	116.06 (14)
C3—C5—Fe1	121.83 (14)	C4—N3—N4	116.73 (15)
C7—C6—C5	107.82 (17)	C4—N3—Zn1	124.85 (12)
C7—C6—Fe1	70.56 (12)	N4—N3—Zn1	118.42 (12)
C5—C6—Fe1	69.44 (11)	C2—N4—N3	114.37 (15)
			
N3—Zn1—S1—C1	−113.37 (8)	C18—Fe2—C15—C16	−81.30 (12)
N1—Zn1—S1—C1	−1.08 (8)	C22—Fe2—C15—C16	159.32 (13)
S2—Zn1—S1—C1	137.52 (6)	C23—Fe2—C15—C16	−165.3 (2)
N3—Zn1—S2—C2	1.28 (7)	C17—Fe2—C15—C16	−37.46 (12)
N1—Zn1—S2—C2	−111.96 (8)	C21—Fe2—C15—C4	0.75 (19)
S1—Zn1—S2—C2	130.25 (6)	C16—Fe2—C15—C4	−114.9 (2)
Zn1—S1—C1—N2	1.38 (18)	C24—Fe2—C15—C4	−75.6 (4)
Zn1—S1—C1—N5	−176.84 (14)	C19—Fe2—C15—C4	126.3 (2)
Zn1—S2—C2—N4	−1.36 (17)	C20—Fe2—C15—C4	−40.8 (2)
Zn1—S2—C2—N6	178.92 (14)	C18—Fe2—C15—C4	163.75 (18)
N1—C3—C5—C6	166.79 (19)	C22—Fe2—C15—C4	44.4 (2)
N1—C3—C5—C9	−19.9 (3)	C23—Fe2—C15—C4	79.7 (3)
N1—C3—C5—Fe1	−109.40 (19)	C17—Fe2—C15—C4	−152.41 (18)
C12—Fe1—C5—C6	43.1 (4)	C21—Fe2—C15—C19	−125.54 (13)
C9—Fe1—C5—C6	−118.84 (16)	C16—Fe2—C15—C19	118.76 (15)
C11—Fe1—C5—C6	76.33 (16)	C24—Fe2—C15—C19	158.1 (3)
C13—Fe1—C5—C6	−167.21 (19)	C20—Fe2—C15—C19	−167.09 (13)
C7—Fe1—C5—C6	−37.81 (12)	C18—Fe2—C15—C19	37.45 (11)
C14—Fe1—C5—C6	159.16 (12)	C22—Fe2—C15—C19	−81.92 (14)
C10—Fe1—C5—C6	117.03 (13)	C23—Fe2—C15—C19	−46.6 (2)
C8—Fe1—C5—C6	−81.26 (12)	C17—Fe2—C15—C19	81.30 (12)
C6—Fe1—C5—C9	118.84 (16)	C4—C15—C16—C17	178.27 (17)
C12—Fe1—C5—C9	161.9 (3)	C19—C15—C16—C17	0.9 (2)
C11—Fe1—C5—C9	−164.83 (15)	Fe2—C15—C16—C17	59.90 (14)
C13—Fe1—C5—C9	−48.4 (2)	C4—C15—C16—Fe2	118.36 (17)
C7—Fe1—C5—C9	81.02 (13)	C19—C15—C16—Fe2	−58.96 (12)
C14—Fe1—C5—C9	−82.01 (14)	C21—Fe2—C16—C17	157.19 (14)
C10—Fe1—C5—C9	−124.13 (13)	C24—Fe2—C16—C17	73.31 (16)
C8—Fe1—C5—C9	37.58 (12)	C19—Fe2—C16—C17	−80.87 (13)
C6—Fe1—C5—C3	−115.6 (2)	C20—Fe2—C16—C17	114.77 (14)
C12—Fe1—C5—C3	−72.5 (4)	C18—Fe2—C16—C17	−37.42 (13)
C9—Fe1—C5—C3	125.6 (2)	C22—Fe2—C16—C17	−168.5 (2)
C11—Fe1—C5—C3	−39.3 (2)	C23—Fe2—C16—C17	40.9 (3)
C13—Fe1—C5—C3	77.2 (3)	C15—Fe2—C16—C17	−119.17 (17)
C7—Fe1—C5—C3	−153.41 (18)	C21—Fe2—C16—C15	−83.64 (14)
C14—Fe1—C5—C3	43.56 (18)	C24—Fe2—C16—C15	−167.52 (12)
C10—Fe1—C5—C3	1.44 (18)	C19—Fe2—C16—C15	38.30 (11)
C8—Fe1—C5—C3	163.15 (18)	C20—Fe2—C16—C15	−126.06 (12)
C9—C5—C6—C7	0.9 (2)	C18—Fe2—C16—C15	81.76 (12)
C3—C5—C6—C7	175.62 (18)	C22—Fe2—C16—C15	−49.4 (3)
Fe1—C5—C6—C7	60.40 (15)	C23—Fe2—C16—C15	160.1 (3)
C9—C5—C6—Fe1	−59.46 (13)	C17—Fe2—C16—C15	119.17 (17)
C3—C5—C6—Fe1	115.23 (18)	C15—C16—C17—C18	−0.8 (2)
C5—Fe1—C6—C7	−118.62 (17)	Fe2—C16—C17—C18	58.79 (14)
C12—Fe1—C6—C7	76.12 (16)	C15—C16—C17—Fe2	−59.63 (13)
C9—Fe1—C6—C7	−80.45 (13)	C21—Fe2—C17—C16	−51.1 (3)
C11—Fe1—C6—C7	118.10 (14)	C24—Fe2—C17—C16	−125.39 (13)
C13—Fe1—C6—C7	44.8 (3)	C19—Fe2—C17—C16	82.81 (13)
C14—Fe1—C6—C7	−168.0 (2)	C20—Fe2—C17—C16	−83.12 (14)
C10—Fe1—C6—C7	159.49 (14)	C18—Fe2—C17—C16	119.90 (18)
C8—Fe1—C6—C7	−36.96 (12)	C22—Fe2—C17—C16	165.1 (3)
C12—Fe1—C6—C5	−165.26 (13)	C23—Fe2—C17—C16	−165.58 (13)
C9—Fe1—C6—C5	38.17 (11)	C15—Fe2—C17—C16	38.20 (12)
C11—Fe1—C6—C5	−123.29 (13)	C21—Fe2—C17—C18	−171.0 (2)
C13—Fe1—C6—C5	163.4 (2)	C16—Fe2—C17—C18	−119.90 (18)
C7—Fe1—C6—C5	118.62 (17)	C24—Fe2—C17—C18	114.71 (13)
C14—Fe1—C6—C5	−49.4 (3)	C19—Fe2—C17—C18	−37.09 (12)
C10—Fe1—C6—C5	−81.89 (15)	C20—Fe2—C17—C18	156.98 (12)
C8—Fe1—C6—C5	81.66 (12)	C22—Fe2—C17—C18	45.2 (4)
C5—C6—C7—C8	−0.6 (2)	C23—Fe2—C17—C18	74.52 (15)
Fe1—C6—C7—C8	59.10 (16)	C15—Fe2—C17—C18	−81.70 (13)
C5—C6—C7—Fe1	−59.69 (14)	C16—C17—C18—C19	0.4 (2)
C5—Fe1—C7—C6	38.62 (12)	Fe2—C17—C18—C19	59.06 (13)
C12—Fe1—C7—C6	−122.82 (13)	C16—C17—C18—Fe2	−58.65 (14)
C9—Fe1—C7—C6	83.19 (13)	C21—Fe2—C18—C19	46.1 (4)
C11—Fe1—C7—C6	−80.12 (14)	C16—Fe2—C18—C19	−82.83 (12)
C13—Fe1—C7—C6	−164.12 (12)	C24—Fe2—C18—C19	157.47 (13)
C14—Fe1—C7—C6	164.3 (3)	C20—Fe2—C18—C19	−169.99 (18)
C10—Fe1—C7—C6	−47.7 (3)	C22—Fe2—C18—C19	75.58 (15)
C8—Fe1—C7—C6	120.23 (17)	C23—Fe2—C18—C19	115.81 (13)
C6—Fe1—C7—C8	−120.23 (17)	C15—Fe2—C18—C19	−38.33 (11)
C5—Fe1—C7—C8	−81.61 (13)	C17—Fe2—C18—C19	−120.03 (17)
C12—Fe1—C7—C8	116.96 (13)	C21—Fe2—C18—C17	166.1 (3)
C9—Fe1—C7—C8	−37.04 (12)	C16—Fe2—C18—C17	37.19 (12)
C11—Fe1—C7—C8	159.65 (13)	C24—Fe2—C18—C17	−82.50 (15)
C13—Fe1—C7—C8	75.65 (15)	C19—Fe2—C18—C17	120.03 (17)
C14—Fe1—C7—C8	44.0 (4)	C20—Fe2—C18—C17	−50.0 (2)
C10—Fe1—C7—C8	−168.0 (2)	C22—Fe2—C18—C17	−164.39 (14)
C6—C7—C8—C9	0.0 (3)	C23—Fe2—C18—C17	−124.16 (13)
Fe1—C7—C8—C9	58.24 (15)	C15—Fe2—C18—C17	81.69 (13)
C6—C7—C8—Fe1	−58.23 (16)	C17—C18—C19—C15	0.2 (2)
C6—Fe1—C8—C7	37.20 (12)	Fe2—C18—C19—C15	59.23 (12)
C5—Fe1—C8—C7	81.97 (13)	C17—C18—C19—Fe2	−59.05 (14)
C12—Fe1—C8—C7	−80.89 (15)	C16—C15—C19—C18	−0.7 (2)
C9—Fe1—C8—C7	120.34 (18)	C4—C15—C19—C18	−177.67 (18)
C11—Fe1—C8—C7	−47.3 (3)	Fe2—C15—C19—C18	−59.48 (13)
C13—Fe1—C8—C7	−123.47 (14)	C16—C15—C19—Fe2	58.78 (12)
C14—Fe1—C8—C7	−164.71 (13)	C4—C15—C19—Fe2	−118.2 (2)
C10—Fe1—C8—C7	163.7 (3)	C21—Fe2—C19—C18	−165.41 (13)
C6—Fe1—C8—C9	−83.13 (13)	C16—Fe2—C19—C18	80.86 (12)
C5—Fe1—C8—C9	−38.37 (12)	C24—Fe2—C19—C18	−47.8 (2)
C12—Fe1—C8—C9	158.78 (13)	C20—Fe2—C19—C18	162.7 (3)
C11—Fe1—C8—C9	−167.6 (2)	C22—Fe2—C19—C18	−123.86 (13)
C13—Fe1—C8—C9	116.19 (14)	C23—Fe2—C19—C18	−81.39 (14)
C7—Fe1—C8—C9	−120.34 (18)	C15—Fe2—C19—C18	119.07 (16)
C14—Fe1—C8—C9	74.95 (16)	C17—Fe2—C19—C18	37.36 (12)
C10—Fe1—C8—C9	43.4 (3)	C21—Fe2—C19—C15	75.51 (15)
C7—C8—C9—C5	0.6 (2)	C16—Fe2—C19—C15	−38.21 (11)
Fe1—C8—C9—C5	58.98 (13)	C24—Fe2—C19—C15	−166.82 (19)
C7—C8—C9—Fe1	−58.41 (15)	C20—Fe2—C19—C15	43.6 (4)
C6—C5—C9—C8	−0.9 (2)	C18—Fe2—C19—C15	−119.07 (16)
C3—C5—C9—C8	−175.03 (19)	C22—Fe2—C19—C15	117.07 (13)
Fe1—C5—C9—C8	−59.92 (14)	C23—Fe2—C19—C15	159.53 (12)
C6—C5—C9—Fe1	58.99 (13)	C17—Fe2—C19—C15	−81.72 (12)
C3—C5—C9—Fe1	−115.1 (2)	C16—Fe2—C20—C21	116.97 (15)
C6—Fe1—C9—C8	80.60 (13)	C24—Fe2—C20—C21	−119.0 (2)
C5—Fe1—C9—C8	118.93 (17)	C19—Fe2—C20—C21	40.4 (4)
C12—Fe1—C9—C8	−47.9 (3)	C18—Fe2—C20—C21	−165.40 (18)
C11—Fe1—C9—C8	163.6 (3)	C22—Fe2—C20—C21	−38.40 (15)
C13—Fe1—C9—C8	−81.87 (15)	C23—Fe2—C20—C21	−81.92 (16)
C7—Fe1—C9—C8	36.93 (13)	C15—Fe2—C20—C21	75.13 (17)
C14—Fe1—C9—C8	−124.39 (13)	C17—Fe2—C20—C21	159.59 (14)
C10—Fe1—C9—C8	−165.21 (13)	C21—Fe2—C20—C24	119.0 (2)
C6—Fe1—C9—C5	−38.33 (11)	C16—Fe2—C20—C24	−124.07 (15)
C12—Fe1—C9—C5	−166.9 (2)	C19—Fe2—C20—C24	159.3 (3)
C11—Fe1—C9—C5	44.6 (4)	C18—Fe2—C20—C24	−46.4 (3)
C13—Fe1—C9—C5	159.20 (12)	C22—Fe2—C20—C24	80.56 (17)
C7—Fe1—C9—C5	−82.00 (13)	C23—Fe2—C20—C24	37.04 (15)
C14—Fe1—C9—C5	116.68 (12)	C15—Fe2—C20—C24	−165.91 (14)
C10—Fe1—C9—C5	75.86 (15)	C17—Fe2—C20—C24	−81.45 (17)
C8—Fe1—C9—C5	−118.93 (17)	C24—C20—C21—C22	0.7 (2)
C6—Fe1—C10—C11	−79.78 (16)	Fe2—C20—C21—C22	59.94 (15)
C5—Fe1—C10—C11	−123.38 (14)	C24—C20—C21—Fe2	−59.23 (15)
C12—Fe1—C10—C11	38.04 (14)	C16—Fe2—C21—C20	−81.35 (15)
C9—Fe1—C10—C11	−165.68 (13)	C24—Fe2—C21—C20	37.87 (14)
C13—Fe1—C10—C11	82.02 (15)	C19—Fe2—C21—C20	−167.81 (13)
C7—Fe1—C10—C11	−45.5 (3)	C18—Fe2—C21—C20	156.2 (3)
C14—Fe1—C10—C11	119.8 (2)	C22—Fe2—C21—C20	118.6 (2)
C8—Fe1—C10—C11	161.0 (3)	C23—Fe2—C21—C20	81.10 (15)
C6—Fe1—C10—C14	160.43 (14)	C15—Fe2—C21—C20	−125.23 (13)
C5—Fe1—C10—C14	116.83 (15)	C17—Fe2—C21—C20	−45.3 (3)
C12—Fe1—C10—C14	−81.74 (16)	C16—Fe2—C21—C22	160.06 (14)
C9—Fe1—C10—C14	74.53 (17)	C24—Fe2—C21—C22	−80.72 (16)
C11—Fe1—C10—C14	−119.8 (2)	C19—Fe2—C21—C22	73.60 (17)
C13—Fe1—C10—C14	−37.76 (15)	C20—Fe2—C21—C22	−118.6 (2)
C7—Fe1—C10—C14	−165.3 (2)	C18—Fe2—C21—C22	37.6 (4)
C8—Fe1—C10—C14	41.2 (4)	C23—Fe2—C21—C22	−37.50 (15)
C14—C10—C11—C12	0.0 (3)	C15—Fe2—C21—C22	116.18 (14)
Fe1—C10—C11—C12	−59.19 (17)	C17—Fe2—C21—C22	−163.9 (2)
C14—C10—C11—Fe1	59.21 (16)	C20—C21—C22—C23	−0.6 (2)
C6—Fe1—C11—C10	117.96 (14)	Fe2—C21—C22—C23	59.54 (15)
C5—Fe1—C11—C10	75.50 (16)	C20—C21—C22—Fe2	−60.10 (15)
C12—Fe1—C11—C10	−119.4 (2)	C21—Fe2—C22—C23	−118.9 (2)
C9—Fe1—C11—C10	41.1 (4)	C16—Fe2—C22—C23	−166.2 (2)
C13—Fe1—C11—C10	−81.20 (15)	C24—Fe2—C22—C23	−37.15 (14)
C7—Fe1—C11—C10	160.21 (13)	C19—Fe2—C22—C23	114.41 (14)
C14—Fe1—C11—C10	−37.31 (14)	C20—Fe2—C22—C23	−81.10 (15)
C8—Fe1—C11—C10	−165.9 (2)	C18—Fe2—C22—C23	73.08 (17)
C6—Fe1—C11—C12	−122.66 (16)	C15—Fe2—C22—C23	157.92 (13)
C5—Fe1—C11—C12	−165.12 (15)	C17—Fe2—C22—C23	38.5 (4)
C9—Fe1—C11—C12	160.5 (3)	C16—Fe2—C22—C21	−47.3 (3)
C13—Fe1—C11—C12	38.17 (16)	C24—Fe2—C22—C21	81.78 (16)
C7—Fe1—C11—C12	−80.41 (18)	C19—Fe2—C22—C21	−126.66 (15)
C14—Fe1—C11—C12	82.07 (17)	C20—Fe2—C22—C21	37.83 (14)
C10—Fe1—C11—C12	119.4 (2)	C18—Fe2—C22—C21	−167.99 (14)
C8—Fe1—C11—C12	−46.6 (3)	C23—Fe2—C22—C21	118.9 (2)
C10—C11—C12—C13	−0.2 (3)	C15—Fe2—C22—C21	−83.15 (16)
Fe1—C11—C12—C13	−60.04 (16)	C17—Fe2—C22—C21	157.4 (3)
C10—C11—C12—Fe1	59.86 (17)	C21—C22—C23—C24	0.2 (2)
C6—Fe1—C12—C13	−165.28 (13)	Fe2—C22—C23—C24	59.20 (16)
C5—Fe1—C12—C13	161.5 (3)	C21—C22—C23—Fe2	−59.00 (14)
C9—Fe1—C12—C13	−47.7 (3)	C21—Fe2—C23—C24	−81.36 (16)
C11—Fe1—C12—C13	118.2 (2)	C16—Fe2—C23—C24	42.7 (3)
C7—Fe1—C12—C13	−123.80 (15)	C19—Fe2—C23—C24	156.97 (14)
C14—Fe1—C12—C13	37.68 (14)	C20—Fe2—C23—C24	−37.59 (15)
C10—Fe1—C12—C13	81.17 (16)	C18—Fe2—C23—C24	114.93 (15)
C8—Fe1—C12—C13	−81.74 (17)	C22—Fe2—C23—C24	−119.6 (2)
C6—Fe1—C12—C11	76.5 (2)	C15—Fe2—C23—C24	−169.61 (18)
C5—Fe1—C12—C11	43.3 (4)	C17—Fe2—C23—C24	73.89 (17)
C9—Fe1—C12—C11	−165.9 (2)	C21—Fe2—C23—C22	38.26 (15)
C13—Fe1—C12—C11	−118.2 (2)	C16—Fe2—C23—C22	162.4 (3)
C7—Fe1—C12—C11	118.01 (17)	C24—Fe2—C23—C22	119.6 (2)
C14—Fe1—C12—C11	−80.51 (18)	C19—Fe2—C23—C22	−83.41 (16)
C10—Fe1—C12—C11	−37.02 (17)	C20—Fe2—C23—C22	82.03 (16)
C8—Fe1—C12—C11	160.07 (16)	C18—Fe2—C23—C22	−125.44 (15)
C11—C12—C13—C14	0.3 (3)	C15—Fe2—C23—C22	−50.0 (3)
Fe1—C12—C13—C14	−59.62 (16)	C17—Fe2—C23—C22	−166.48 (14)
C11—C12—C13—Fe1	59.89 (16)	C22—C23—C24—C20	0.2 (3)
C6—Fe1—C13—C12	41.2 (3)	Fe2—C23—C24—C20	59.54 (15)
C5—Fe1—C13—C12	−166.20 (19)	C22—C23—C24—Fe2	−59.29 (15)
C9—Fe1—C13—C12	158.90 (15)	C21—C20—C24—C23	−0.6 (2)
C11—Fe1—C13—C12	−38.52 (17)	Fe2—C20—C24—C23	−59.79 (16)
C7—Fe1—C13—C12	75.27 (17)	C21—C20—C24—Fe2	59.19 (15)
C14—Fe1—C13—C12	−119.1 (2)	C21—Fe2—C24—C23	81.92 (17)
C10—Fe1—C13—C12	−81.65 (17)	C16—Fe2—C24—C23	−164.90 (14)
C8—Fe1—C13—C12	116.47 (15)	C19—Fe2—C24—C23	−48.7 (3)
C6—Fe1—C13—C14	160.4 (2)	C20—Fe2—C24—C23	119.7 (2)
C5—Fe1—C13—C14	−47.1 (3)	C18—Fe2—C24—C23	−81.88 (16)
C12—Fe1—C13—C14	119.1 (2)	C22—Fe2—C24—C23	37.39 (15)
C9—Fe1—C13—C14	−81.96 (15)	C15—Fe2—C24—C23	164.2 (3)
C11—Fe1—C13—C14	80.62 (16)	C17—Fe2—C24—C23	−124.39 (15)
C7—Fe1—C13—C14	−165.59 (14)	C21—Fe2—C24—C20	−37.75 (16)
C10—Fe1—C13—C14	37.48 (15)	C16—Fe2—C24—C20	75.43 (18)
C8—Fe1—C13—C14	−124.39 (14)	C19—Fe2—C24—C20	−168.37 (18)
C11—C10—C14—C13	0.2 (3)	C18—Fe2—C24—C20	158.45 (14)
Fe1—C10—C14—C13	59.29 (16)	C22—Fe2—C24—C20	−82.28 (17)
C11—C10—C14—Fe1	−59.14 (17)	C23—Fe2—C24—C20	−119.7 (2)
C12—C13—C14—C10	−0.3 (3)	C15—Fe2—C24—C20	44.5 (4)
Fe1—C13—C14—C10	−59.58 (16)	C17—Fe2—C24—C20	115.94 (16)
C12—C13—C14—Fe1	59.32 (17)	C5—C3—N1—N2	178.99 (17)
C6—Fe1—C14—C10	−45.4 (3)	C5—C3—N1—Zn1	−6.0 (3)
C5—Fe1—C14—C10	−81.29 (17)	N3—Zn1—N1—C3	−47.56 (19)
C12—Fe1—C14—C10	81.40 (16)	S1—Zn1—N1—C3	−173.83 (17)
C9—Fe1—C14—C10	−124.68 (15)	S2—Zn1—N1—C3	55.77 (19)
C11—Fe1—C14—C10	36.99 (15)	N3—Zn1—N1—N2	127.37 (11)
C13—Fe1—C14—C10	119.2 (2)	S1—Zn1—N1—N2	1.10 (11)
C7—Fe1—C14—C10	160.6 (3)	S2—Zn1—N1—N2	−129.30 (10)
C8—Fe1—C14—C10	−165.85 (14)	N5—C1—N2—N1	177.48 (16)
C6—Fe1—C14—C13	−164.60 (19)	S1—C1—N2—N1	−0.7 (3)
C5—Fe1—C14—C13	159.54 (12)	C3—N1—N2—C1	175.39 (17)
C12—Fe1—C14—C13	−37.77 (14)	Zn1—N1—N2—C1	−0.54 (19)
C9—Fe1—C14—C13	116.15 (14)	C15—C4—N3—N4	1.1 (3)
C11—Fe1—C14—C13	−82.18 (15)	C15—C4—N3—Zn1	−179.14 (14)
C7—Fe1—C14—C13	41.4 (4)	N1—Zn1—N3—C4	−48.03 (16)
C10—Fe1—C14—C13	−119.2 (2)	S1—Zn1—N3—C4	53.21 (16)
C8—Fe1—C14—C13	74.98 (17)	S2—Zn1—N3—C4	178.69 (15)
N3—C4—C15—C16	167.78 (18)	N1—Zn1—N3—N4	131.69 (12)
N3—C4—C15—C19	−15.6 (3)	S1—Zn1—N3—N4	−127.08 (11)
N3—C4—C15—Fe2	−107.2 (2)	S2—Zn1—N3—N4	−1.59 (12)
C21—Fe2—C15—C16	115.70 (13)	N6—C2—N4—N3	−179.90 (15)
C24—Fe2—C15—C16	39.4 (4)	S2—C2—N4—N3	0.4 (2)
C19—Fe2—C15—C16	−118.76 (15)	C4—N3—N4—C2	−179.13 (16)
C20—Fe2—C15—C16	74.16 (15)	Zn1—N3—N4—C2	1.13 (19)

### Hydrogen-bond geometry (Å, °)

**Table:**

*D*—H···*A*	*D*—H	H···*A*	*D*···*A*	*D*—H···*A*
N5—H2N5···S2^i^	0.80 (3)	2.80 (2)	3.4880 (19)	146 (2)
N6—H2N6···N2^ii^	0.81 (3)	2.21 (3)	2.983 (2)	161 (3)
C7—H7A···N2^iii^	0.98	2.56	3.465 (3)	153
C9—H9A···S2	0.98	2.73	3.697 (2)	168
C17—H17A···S2^iv^	0.98	2.70	3.479 (2)	137
C19—H19A···N4	0.98	2.51	2.936 (2)	106
N5—H1N5···Cg1^v^	0.90 (3)	2.85 (3)	3.515 (2)	132 (2)
C6—H6A···Cg2^vi^	0.98	2.80	3.755 (2)	164
C9—H9A···Cg3	0.98	2.47	3.247 (2)	135
C10—H10A···Cg3^vi^	0.98	2.93	3.674 (3)	134
C24—H24A···Cg4^iv^	0.98	2.83	3.629 (2)	139

Symmetry codes: (i) −*x*+1/2, *y*+1/2, −*z*+1/2; (ii) *x*−1/2, −*y*+3/2, *z*−1/2; (iii) −*x*+3/2, *y*−1/2, −*z*+1/2; (iv) *x*+1/2, −*y*+3/2, *z*−1/2; (v) −*x*+3/2, *y*+1/2, −*z*+1/2; (vi) *x*+1/2, −*y*+3/2, *z*+1/2.
